# Self-Interest Induces Counter- Empathy at the Late Stage of Empathic Responses to Others’ Economic Payoffs

**DOI:** 10.3389/fpsyg.2019.00372

**Published:** 2019-02-25

**Authors:** Jing Jie, Pinchao Luo, Mengdi Zhuang, Beibei Li, Yu Pang, Junjiao Li, Xifu Zheng

**Affiliations:** ^1^Guangdong Key Laboratory of Mental Health and Cognitive Science, School of Psychology, Center for Studies of Psychological Application, South China Normal University, Guangzhou, China; ^2^Center for Mental Health Education, Hainan University, Haikou, China

**Keywords:** empathy, fairness preference, event-related potential (ERP), N170, late positive potential (LPP), feedback-related negativity (FRN)

## Abstract

Previous studies have found that individuals exhibit empathic responses when others are treated unfairly. However, there remains a lack of clarity over the extent to which self-interest regulates these empathic responses, and in identifying which component of empathy is more likely to be affected. To investigate these issues, an experiment was designed based on a money distribution task with two conditions [observation condition (OC) vs. participation condition (PC)], and carried out using scalp-recorded event-related potentials (ERPs). Behavioral data showed that the participants’ empathic responses were consistent with their coplayers’ emotional expressions in the OC, whereas they were inconsistent with the coplayers’ expressions in the PC. The electrophysiological data showed that the neural encoding of facial expressions (reflected in the N170) was not affected by self-interest. However, the late stage of empathic responses (LPP) showed a decline when participants’ self-interest was involved. Disadvantageous inequality and relatively fair distribution to others elicited a more pronounced feedback-related negativity (FRN) than advantageous inequality distribution in both the OC and PC. As the late stage of empathic responses is also indexed by the LPP amplitude, these results indicate that the participants were more concerned for their own outcomes than for others’ benefits when self-interest was involved, which reduced their empathy toward their coplayers at the late stage of empathic responses.

## Introduction

Empathy is an important contributor to successful social interaction and a key motivator for altruistic behavior, allowing us to predict and understand others’ behavior and to react accordingly. As social creatures, we are able not only to infer the intentions of others’ behaviors, but also to share the mental and emotional states of others due to our capability for empathy (Decety and Lamm, 2006; de Vignemont and Singer, 2006; Singer, 2006; Frith and Singer, 2008; Hein and Singer, 2008; Singer and Lamm, 2009; Kirman and Teschl, 2010; Cuff et al., 2016). Surprisingly, however, most studies to date have focused on empathy for physical pain rather than empathy for negative social experiences, such as unfair treatment, even though observing the latter is likely to be a more frequent occurrence. Numerous studies on the neural bases of empathy have shown the anterior insula (AI) and mid-anterior cingulate cortex (mACC) to be implicated in first-hand and vicarious experiences of pain (Singer et al., 2004; Farrell et al., 2005; Salimi-Khorshidi et al., 2009; Corradi-Dell’Acqua et al., 2011; Lamm et al., 2011); disgust (Murphy et al., 2003; Wicker et al., 2003; Jabbi et al., 2007; Vytal and Hamann, 2010); and unfairness (Sanfey et al., 2003; Civai et al., 2012; Corradi-Dell’Acqua et al., 2013; Gabay et al., 2014; Feng et al., 2015). The findings of these studies suggest that we feel unpleasant when unfair treatment is meted out to ourselves or to others. These results have provided support for the inequity aversion and fairness preference in Ultimatum Games (UG). The findings from the UG task indicated that the participants respond to a proposer’s unfair offers by lowering both their own and others’ payoffs (Charness and Rabin, 2002; Sanfey et al., 2003). That is, our empathy for others may promote altruistic behavior, a preference for fairness, and disgust toward unfair transactions or distributions.

However, in real life, we usually tend to help others generously when our own interests are not involved. It is difficult for most people to make every effort to sacrifice their own interests to help others. This may be because, when self-interest is involved, we are more concerned about our own interest, resulting in reduced empathic responses toward others. Previous studies have confirmed empathic responses are not adaptive to all social interactions, being modulated by contextual appraisal (Gu and Han, 2007; Hein and Singer, 2008; Singer and Lamm, 2009; Yamada et al., 2011). For example, Yamada et al. (2011) showed that congruence (in the cooperative setting) between the co-player’s affective expression and the participant’s outcome (win or loss) enhanced initial empathic responses, whereas incongruence (in the competitive setting) led to counter-empathic responses. These results showed that empathic responses can be reduced by a competitive relationship.

Even if there is no direct competition, we are also likely to exhibit incongruent emotions toward others once self-interest is involved. In an electrophysiological study, observers evaluated the outcomes for their friends versus strangers in a gambling task (Ma et al., 2011). The discrepancy of the FRN loss–win difference wave (d-FRN) between the outcomes for friends and strangers could only be observed when the observer did not participate in the gamble. These results indicate that the participants experienced more motivational relevance toward their friends than to strangers, but also that the participants’ empathic responses toward friends were only salient when they were not directly involved in the gamble. In other words, we are more concerned about our own outcomes compared with others’ benefits (Fukushima and Hiraki, 2006; Itagaki and Katayama, 2008). We are used to evaluating others’ outcomes from an egocentric perspective. The larger the extent to which self-benefit is involved, the more likely it is that egoism will influence one’s affective response toward others’ monetary outcomes (Wang et al., 2014a). The stronger empathy for a friend compared to a stranger may also be because the friend is self-related, in that the participants may have selfish incentives for increasing the chance of future cooperative interactions.

In reviewing previous empathy research, it is evident that there are still some issues that are unclear. How does the involvement of self-interest regulate empathic responses toward others who suffer unfair treatment? It has been suggested that empathy involves both an early automatic component characterized by emotional sharing (bottom-up processing) and a late controlled component characterized by cognitive evaluation (top-down processing) (Decety and Lamm, 2006; Shamay-Tsoory et al., 2007, 2009; Xiang et al., 2018). This raises the question, ‘Which component is more likely to be affected by self-interest?’ Addressing this issue could help to resolve some of the gaps and apparent anomalies in the current literature on empathy.

In an attempt to resolve these issues, the current study used scalp-recorded event-related potentials (ERPs) which provide a continuous measure of processing between a stimulus and a response, making it possible to determine which stage(s) of empathy are being affected by our experimental manipulation. We designed a money distribution task with two conditions [observation condition (OC) vs. participation condition (PC)], adapted from the classic Dictator Game (Engel, 2011). In the OC, the participants took part in the game as observers and observed a proposer allocating money to a coplayer. In the PC, the proposer allocated money to the participant and the coplayer. The EEGs from the participants were recorded simultaneously. There were three kinds of money distribution to the coplayer, as shown in Table 1: disadvantageous inequality (DI, in which the coplayer receives less money than the proposer or the participant), relatively fair (RF; the coplayer’s share is basically equal to that of the others); and advantageous inequality (AI, the coplayer receives more money than the proposer or the participant).

**Table 1:** The list of payoff pairs used in the money distribution task.

	OC		PC	
	Proposer	Coplayer	Self	Coplayer
DI	9	1	9	1
	8	2	8	2
RF	6	4	6	4
	5	5	5	5
	4	6	4	6
AI	2	8	2	8
	1	9	1	9

*The unit is ¥. OC, observation condition; PC, participation condition; DI, disadvantageous inequality; RF, relatively fair; AI, advantageous inequality.*

Our analyses focused on three distinct ERP components. The first component was N170, a face-sensitive ERP component (Caharel et al., 2009) characterized by a negative deflection in wave amplitude that occurs around 150–200 ms over occipito-temporal sites (Bentin et al., 1996; Maurer et al., 2005). A study has demonstrated a relationship between the magnitude of N170 and the occurrence of spontaneous facial mimicry (Achaibou et al., 2008). Mimicry is an ability to adopt the emotional state of another person, which can occur automatically and has been designated as the most basic form of empathy (Christov-Moore et al., 2014). This means that N170 is associated with the early stage of empathy (the bottom-up process of emotional sharing). As a targeted component in the current study, N170 was used to investigate whether self-interest influences the early stage of empathy. The second component of ERPs, called the late positive potential (LPP), is a centro-parietal positive deflection that usually appears between 300 and 700 ms, reflecting a long latency empathic response (Fan and Han, 2008). Through the use of LPP, we can examine whether self-interest affects the later stages of empathy. The third component of ERPs is called feedback-related negativity (FRN). FRN is an ERP component that peaks at about 250 ms after the onset of the outcome feedback, and is more prominent for stimuli with unfavorable outcomes as opposed to favorable outcomes (Gehring and Willoughby, 2002; Holroyd and Coles, 2002; Nieuwenhuis et al., 2005; Holroyd et al., 2006; Masaki et al., 2006; Hajcak et al., 2007; Yu et al., 2007; Walsh and Anderson, 2012). Through the FRN, we can identify with greater accuracy the point in time when self-interest affects empathy.

Based on previous evidence, we predict that involvement of self-interest could weaken empathic responses toward those who suffer unfair treatment. If there are differences in the amplitude of N170 between the OC and PC, this indicates that self-interest has affected the neural encoding of facial expressions. This could interfere with the emotional resonance with others; that is to say, self-interest could affect the early stage of empathic responses to economic inequality (i.e., emotional sharing). If there are no significant differences, this indicates on the contrary that self-interest has not affected the recognition of facial expressions, and therefore has not weakened emotional resonance. If there are differences in the LPP amplitude between the OC and PC, this indicates that self-interest has affected the late stage of empathy; the absence of significant differences indicates that self-interest has not affected the late stage of empathy. If the FRN amplitude in the disadvantageous inequality situation is higher than in the advantageous inequality situation under both the OC and PC, this indicates that the participants viewed the outcome of money distribution from the coplayer’s perspective (i.e., an empathic response) at the beginning of empathic responses. If the FRN amplitude shows the opposite trend under both conditions, this indicates that the participants viewed the outcome from their own perspective (a counter-empathic response) at the beginning of empathic responses.

At the behavioral level, we predicted that the participants would exhibit opposite emotional responses to the others’ facial expressions in the OC compared to the PC. More specifically, the participants’ emotional responses should be emotionally congruent with the coplayer in the OC, and emotionally incongruent with the coplayer in the PC.

## Materials and Methods

### Participants

Twenty-six participants were recruited to this study (13 women, 13 men; mean age: 20.46 years ± 2.14 SD). All participants were college students, right handed, had normal or corrected to normal vision, and did not have any history of neurological or mental disorders. This study was carried out in accordance with the recommendations of Ethics Committee of South China Normal University. All participants provided informed written consent before taking part in the experiment and were paid for their participation. The procedure of the experiment was consistent with the principles of international research involving human subjects as stated in the Declaration of Helsinki (World Medical Organization, 1999). The study was approved by the Ethics Committee of South China Normal University.

### Visual Stimuli

Participants were comfortably seated in a dimly lit, sound-attenuated, and electrically shielded room. The stimuli were presented at the center of a 17 inch color monitor with a white background. Each stimulus (inclusion of text information) was a 10.5 cm × 14 cm (width × height) picture, subtending a visual angle of 6.02° × 8.02° at a viewing distance of 100 cm. As an observer, the participant saw a proposer giving an initial endowment of 10 yuan to a coplayer of the game. As a participant, he or she saw the proposer allocating 10 yuan to himself or herself and the coplayer. The facial expressions of the male or female proposer (gender-matched) serving during the experiment were neutral and selected from an ID photo. Feedback consisted of three kinds of facial expressions (frown, neutral, and smile) of the male or female coplayer (gender-matched), selected from the CAS-PEAL Face Database (Wen et al., 2008). Three still shots were used, clearly expressing a discernible frown, neutral expression, and smile, in response to a disadvantageous inequality (DI) distribution, relatively fair (RF) distribution, and advantageous inequality (AI) distribution to the coplayer, respectively. Brightness, size, contrast, and color settings of these pictures were unified with photo editing software.

### Experimental Procedure

Before recording, participants read the instructions and the rules of the experimental task. We used a payoff distribution task preceded by an Ultimatum Game (UG; Sanfey et al., 2003). The participants were asked to perform a classical UG task for 1 min. The aim of the task was to encourage the participants to empathize with others, thereby enhancing the ecological validity of the formal experiment. In each trial of the UG task, the participants faced an unknown proposer who made an offer on how to divide an initial endowment of 10 yuan. The participants could accept the offer or reject it. Participants were instructed to use the keypad to make their choices. Data with respect to the UG were not relevant to the current study and thus not recorded. At the end of this session, participants were asked two questions, as follows. The first was, “As an observer, what are your feelings if your coplayer receives less money?” (1 = “unpleasant”, 2 = “pleasant”, 3 = “no feeling”); and the second was, “As an observer, what are your feelings if your coplayer receives more money?” (1 = “unpleasant,” 2 = “pleasant,” 3 = “no feeling”). All of the participants’ answers to the questions were “unpleasant” and “pleasant,” respectively. This suggests that the participants responded empathically toward the coplayer in relation to the money distribution outcome. Following the UG task, we then conducted the formal experiment, including the OC and PC.

In the OC, the participants were convinced that the proposer was empowered to distribute 10 yuan to the coplayer. As observers, the participants did not have the chance to receive a payment of 10 yuan. Table 1 shows the offers that were defined *a priori* by the experimenters. These ranged from extreme DI (distribution ratio of 1:9 or 2:8, where the proposer offered 1 or 2 yuan out of 10 yuan to the coplayer, leaving the rest to himself/herself), to RF (distribution ratio of 5:5, 6:4, or 4:6), and AI (distribution ratio of 9:1 or 8:2). Each observer was asked to pay attention to the facial expression of the coplayer and the outcome of the distribution. The three levels of fairness were randomly presented.

**FIGURE 1:**
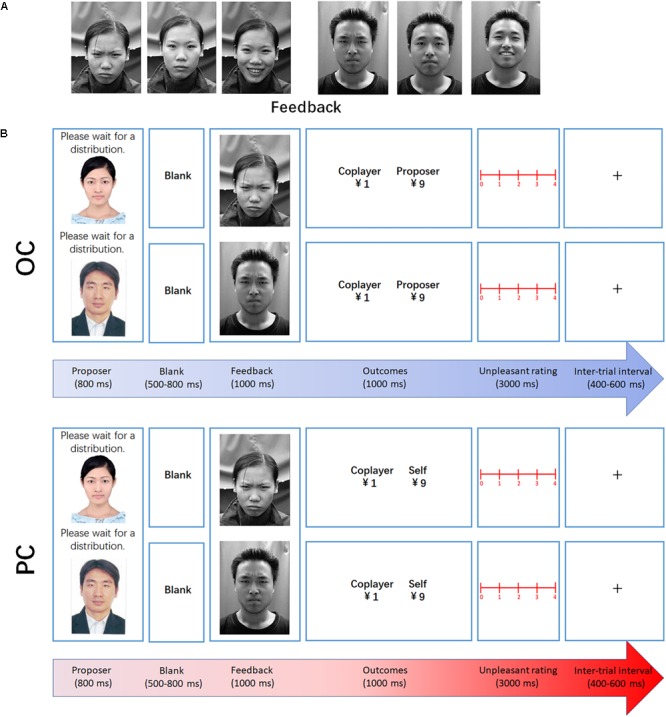
Experimental design. **(A)** Examples of the facial expressions used in the experiment (feedback: frown, neutral, and smile, in response to DI, RF, and AI distribution to the coplayer, respectively). **(B)** Examples of DI trials in the observation and participation conditions. At the beginning of each trial, a fixation mark was displayed at the center of the screen for a duration of 400–600 ms. After the fixation marker, the participants were presented with the proposer’s photograph for 800 ms, followed by a 500–800 ms blank screen prior to the onset of the coplayer’s facial expression. Subsequently, the financial outcome was presented for 1000 ms.

At the beginning of each trial, a fixation mark was displayed at the center of the screen for a duration of 400–600 ms. After the fixation marker, the participants were presented with the proposer’s photograph for 800 ms, followed by a 500–800 ms blank screen prior to the onset of the coplayer’s facial expression (frown, neutral, and smile, in response to DI, RF, and AI distribution to the coplayer, respectively). The facial expression remained on screen for 1000 ms. Subsequently, the financial outcome was presented for 1000 ms (see Figure 1). Each level of fairness was presented for 60 trials, making a total of 180 trials. To encourage the participants to concentrate on the task, a question mark was presented instead of the outcome presentation every 30 trials. The participants were asked to judge whether the money offered to the coplayer was AI, RF, or DI at this time. The keyboard numbers 1, 2, and 3 represented AI, RF, and DI, respectively. At the end of the OC task, the participants completed a rating scale to measure the degree of unpleasantness experienced for each level of fairness, using a 5-point scale ranging from 0 (“not at all”) to 4 (“very much”). Each distribution ratio (1:9, 2:8, 4:6, 5:5, 6:4, 8:2, and 9:1) was presented once.

In the PC, the proposers were empowered to distribute 10 yuan to the participants and the coplayer. As shown in Table 1, the offers were defined a priori by the experimenters, and were identical to those in the OC. Each level of fairness was presented for 60 trials, making a total of 180 trials. The participants were told that one trial from the pool of money distribution would be drawn at random as their reward.

In summary, there were 400 trials in the whole experiment, including 14 practice trials, 12 judgment trials, and 14 rating trials. Note that only 360 trials of the formal experiment were analyzed; the judgment and assessment data were not included. The participants took part in the game firstly as observers and subsequently participated in the money distribution task.

### EEG Recording and Analysis

The electroencephalogram (EEG) was recorded continuously from 62 scalp electrodes and the addition of two mastoid electrodes with Brain Amp DC amplifiers (Brain Products, Germany). All electrodes were re-referenced offline to an averaged mastoid reference, with a forehead ground being employed. Eye blinks and vertical eye movements were monitored with electrodes located above the right eye. The horizontal EOG (HEOG) recording electrodes were positioned at the outer canthi of both eyes. EEG and EOG activity was amplified by applying a bandpass filter from 0.01 to 100 Hz and was continuously sampled at 500 Hz/channel. All electrode impedances were maintained at <5 kΩ. The EEGs averaged for the trials under each condition were computed separately offline using BrainVision Analyzer 2.0 software (Brain Products, Germany; Fritsch and Kuchinke, 2013). Each epoch continued for 1,200 ms, with 200 ms before the facial expression (feedback) onset for the baseline correction. In addition, we used independent component analysis (ICA) to remove artifacts. Trials contaminated by eye blinks, eye movements, or muscle potentials exceeding ±100 μV at any electrode were excluded from averaging. The total rejection rate of the trials was 2.49%.

Our analyses focused on three distinct ERP components: N170, LPP, and FRN. As explained in the introduction, the reasons for choosing these three components can be summarized as follows: N170 is associated with the neural encoding of facial expressions; LPP is sensitive to changes in perceived emotional intensity; and the FRN component is well-established to distinguish between one’s own positive and negative outcomes (San Martin, 2012). Based on previous studies and visual observation, we analyzed the mean amplitude from 140 to 180 ms after the onset of the facial expression for N170; from 330 to 550 ms after the onset of the facial expression for LPP; and from 250 to 350 ms after the onset of the facial expression for FRN. For statistical analysis, we selected two electrodes (P7 and P8) in the occipito-temporal area for N170; nine electrodes (C3, C4, P3, P4, Cz, Pz, CPz, CP3, and CP4) in the central-parietal area for LPP; and nine electrodes (F3, F4, C3, C4, Fz, Cz, FCz, FC3, and FC4) in the fronto-central area for FRN.

The within-subject repeated measures ANOVA was performed with three factors: condition (OC and PC), level of fairness (DI, RF, and AI), and electrodes for N170, LPP, and FRN. The Greenhouse–Geisser correction was applied for the violation of the sphericity assumption in ANOVA where appropriate, and the Bonferroni correction was used for multiple comparisons.

## Results

### Behavioral Data

**FIGURE 2:**
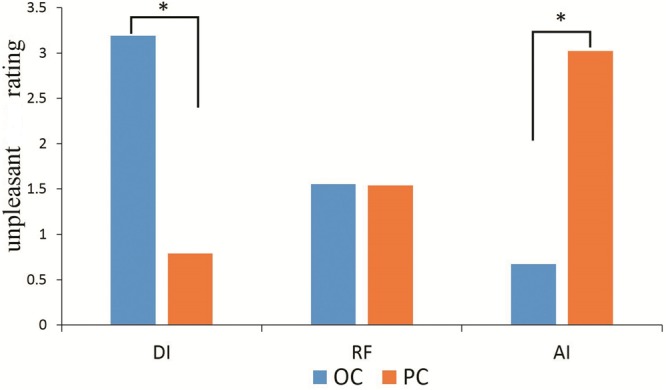
The self-reported unpleasant ratting in each condition (OC-DI, OC-RF, OC-AI, PC-DI, PC-RF, and PC-AI). ^∗^*P* < 0.05.

Figure 2 shows the average unpleasantness rating in each condition (OC-DI, OC-RF, OC-AI, PC-DI, PC-RF, and PC-AI). Repeated-measures analysis of variance (ANOVA) was conducted for the behavioral data, with condition (OC/PC) and fairness (DI/RF/AI) as two independent factors. The main effects for condition [*F*_(1,25)_ = 0.030, *p* > 0.05, $\eta_{p}^{2}$ = 0.001] and fairness [*F*_(2,50)_ = 2.426, *p* > 0.05, $\eta_{p}^{2}$ = 0.088] were not significant. However, the interaction effect between condition and fairness was significant [*F*_(2,50)_ = 89.107, *p* < 0.001, $\eta_{p}^{2}$ = 0.781].

Further simple effect analysis revealed that in the OC, participants rated themselves as feeling more unpleasant in the DI situation (3.192 ± 0.176) than in the AI [0.673 ± 0.149; *t*_(25)_ = 10.455, *p* < 0.001], and RF situations [1.551 ± 0.162; *t*_(25)_ = 7.285, *p* < 0.001]. They also felt more unpleasant in the RF than in the AI situation [*t*_(25)_ = 3.343, *p* < 0.01]. In the PC, however, the participants rated themselves as feeling more unpleasant in the AI situation (3.019 ± 0.219) than in the DI [0.788 ± 0.222; *t*_(25)_ = 6.519, *p* < 0.001] and RF situations [1.538 ± 0.165; *t*_(25)_ = 5.454, *p* < 0.001]. Additionally, they felt more unpleasant in the RF than in the DI situation [*t*_(25)_ = 2.705, *p* < 0.05].

The participants rated themselves in the DI situation as feeling more unpleasant in the OC than in the PC [*t*_(25)_ = 8.012, *p* < 0.001]. In contrast, in the AI situation they felt more unpleasant in the PC than in the OC [*t*_(25)_ = 8.469, *p* < 0.001]. There was no significant difference between the OC and PC in the assessment of unpleasantness in the RF situation (*p* > 0.05).

### ERP Data

#### N170

Supplementary Figures S1, S2 show the grand-averaged ERPs and topographic maps for the N170 amplitudes in different situations. The statistical results for grand-averaged ERPs of N170 with three factors (condition, fairness, and electrode) revealed no significant differences. The main effect for condition [*F*_(1,25)_ = 1.057, *p* > 0.05, $\eta_{p}^{2}$ = 0.041], fairness [*F*_(2,50)_ = 2.736, *p* > 0.05, $\eta_{p}^{2}$ = 0.099] and electrode [*F*_(1,25)_ = 0.838, *p* > 0.05, $\eta_{p}^{2}$ = 0.032] were not significant. The three-way interaction of condition, fairness and electrode was not significant [*F*_(2,50)_ = 0.144, *p* > 0.05, $\eta_{p}^{2}$ = 0.006]. The interaction effect between condition and fairness [*F*_(2,50)_ = 0.521, *p* > 0.05, $\eta_{p}^{2}$ = 0.020], condition and electrode [*F*_(1,25)_ = 0.070, *p* > 0.05, $\eta_{p}^{2}$ = 0.003], fairness and electrode [*F*_(2,50)_ = 0.020, *p* > 0.05, $\eta_{p}^{2}$ = 0.001] were not significant. Table 2 summarizes the ANOVA results for the N170 amplitudes (140–180 ms) with the condition (OC and PC), level of fairness (DI, RF, and AI), and electrode distribution as the within-subject factors. These results indicated that the neural encoding of facial expressions was not affected by self-interest. Therefore, self-interest could not have interfered with the emotional resonance with others. In particular, self-interest did not affect the early stage of empathic responses to economic inequality (i.e., emotional sharing).

**Table 2:** Summary of ANOVA results for the N170 amplitudes (140–180 ms) with the condition (OC and PC), level of fairness (DI, RF, and AI), and electrode distribution as the within-subject factors.

Effect	*F*	*p*	$\eta_{p}^{2}$
Condition	1.057	0.314	0.041
Level of fairness	2.736	0.081	0.099
Electrode distribution	0.838	0.369	0.032
Condition × level of fairness	0.521	0.574	0.020
Condition × electrode distribution	0.070	0.794	0.003
Level of fairness × electrode distribution	0.020	0.977	0.001
Condition × level of fairness × electrode distribution	0.144	0.865	0.006

*OC, observation condition; PC, participation condition; DI, disadvantageous inequality; RF, relatively fair; AI, advantageous inequality*.

#### Late Positive Potential (LPP)

**FIGURE 3:**
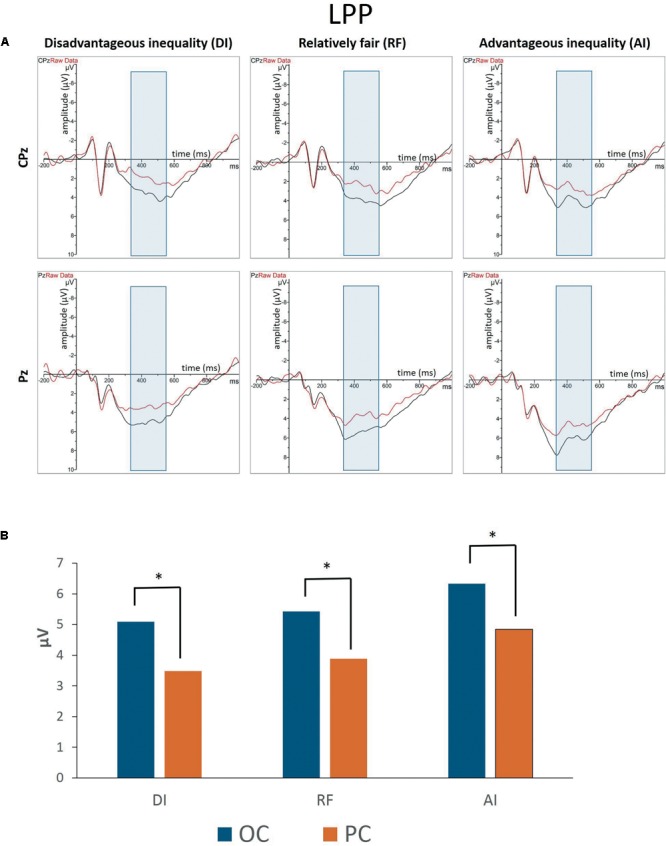
**(A)** Grand-averaged event-related brain potentials (ERPs) for LPP amplitudes in different situations from the CPz and Pz regions. **(B)** The bar graphs show the mean value of the LPP amplitude in each condition at Pz. ^∗^*P* < 0.05.

Figure 3A, 4 show grand-averaged ERPs and topographic maps for LPP amplitudes in different situations. The statistical results for grand-averaged ERPs of LPP with three factors (condition, fairness, and electrode) revealed main effects for condition [*F*_(1,25)_ = 11.936, *p* < 0.01, $\eta_{p}^{2}$ = 0.323], with fairness and electrode being significant [fairness: *F*_(2,50)_ = 6.341, *p* < 0.01, $\eta_{p}^{2}$ = 0.202; electrode: *F*_(8,200)_ = 10.552, *p* < 0.001, $\eta_{p}^{2}$ = 0.824]. Specifically, the OC (*M*_OC-DI_ = 3.632, *SE*_OC-DI_ = 0.563; *M*_OC-RF_ = 3.788, *SE*_OC-RF_ = 0.601; *M*_OC-AI_ = 4.403, *SE*_OC-AI_ = 0.639) elicited significantly enhanced LPP amplitude compared to PC (*M*_PC-DI_ = 2.247, *SE*_PC-DI_ = 0.474; *M*_PC-RF_ = 2.729, *SE*_PC-RF_ = 0.528; *M*_PC-AI_ = 3.240, *SE*_PC-AI_ = 0.557) in all levels of fairness (see Figure 3B). The statistically significant differences were, respectively, as follows: *t*_DI(25)_ = 4.414, *p*_DI_ < 0.001; *t*_RF(25)_ = 2.144, *p*_RF_ < 0.05; and *t*_AI(25)_ = 2.951, *p*_AI_ < 0.01. In sum, the results showed that the grand-averaged ERP of the LPP was larger for the OC than for the PC, regardless of the level of fairness. *Post hoc* pairwise comparisons with Bonferroni correction showed the comparisons of AI (*M*_AI_ = 3.822, *SE*_AI_ = 0.566) with DI (*M*_DI_ = 2.940, *SE*_DI_ = 0.596) and RF (*M*_RF_ = 3.258, *SE*_RF_ = 0.509) were significant [*t*_AI,DI(25)_ = 3.336, *p*_AI,DI_ < 0.01; *t*_AI,RF(25)_ = 2.364, *p*_AI,RF_ < 0.05], whereas the comparison between RF and DI was not.

**FIGURE 4:**
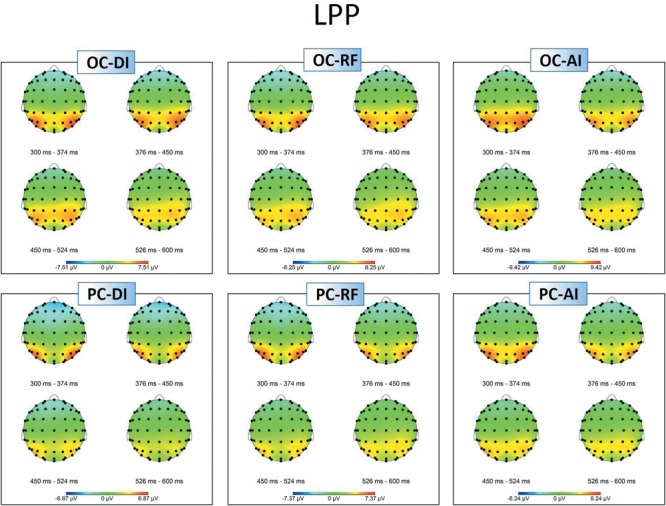
Topographic maps of the LPP amplitudes in different situations.

In addition, compared with the electrodes of Cz (*M* = 1.146, *SE* = 0.712), C3 (*M* = 1.139, *SE* = 0.620), C4 (*M* = 1.854, *SE* = 0.625), CPz (*M* = 3.270, *SE* = 0.633), CP3 (*M* = 3.120, *SE* = 0.524), and CP4 (*M* = 3.633, *SE* = 0.550), more pronounced LPPs were observed at Pz (*M* = 4.845, *SE* = 0.530), P3 (*M* = 5.507, *SE* = 0.515), and P4 (*M* = 5.545, *SE* = 0.498). The interaction effect between condition and electrode was marginal significant [*F*_(8,200)_ = 2.541, *p* = 0.048, $\eta_{p}^{2}$ = 0.530]. Specifically, the P4 amplitudes in the OC (*M* = 6.088, *SE* = 0.521) were higher than those of P3 (*M* = 5.979, *SE* = 0.591), *t*_(25)_ = 0.286, *p* > 0.05. However, the P4 amplitudes in the PC (*M* = 5.003, *SE* = 0.526) were lower than those of P3 (*M* = 5.036, *SE* = 0.507), *t*_(25)_ = -0.093, *p* > 0.05. The Cz amplitudes in the OC (*M* = 1.815, *SE* = 0.795) were higher than those of C3 (*M* = 1.636, *SE* = 0.672), *t*_(25)_ = 0.686, *p* > 0.05. In the PC, however, the Cz amplitudes (*M* = 0.477, *SE* = 0.687) were lower than those of C3 (*M* = 0.642, *SE* = 0.604), *t*_(25)_ = -0.702, *p* > 0.05. The CPz amplitudes in the OC (*M* = 4.035, *SE* = 0.716) were higher than those of CP3 (*M* = 3.614, *SE* = 0.603), *t*_(25)_ = 1.618, *p* > 0.05, whereas the CPz amplitudes in the PC (*M* = 2.506, *SE* = 0.617) were lower than those of CP3 (*M* = 2.626, *SE* = 0.494), *t*_(25)_ = -0.478, *p* > 0.05. The interaction effects of other variables were not significant.

#### Feedback-Related Negativity (FRN)

**FIGURE 5:**
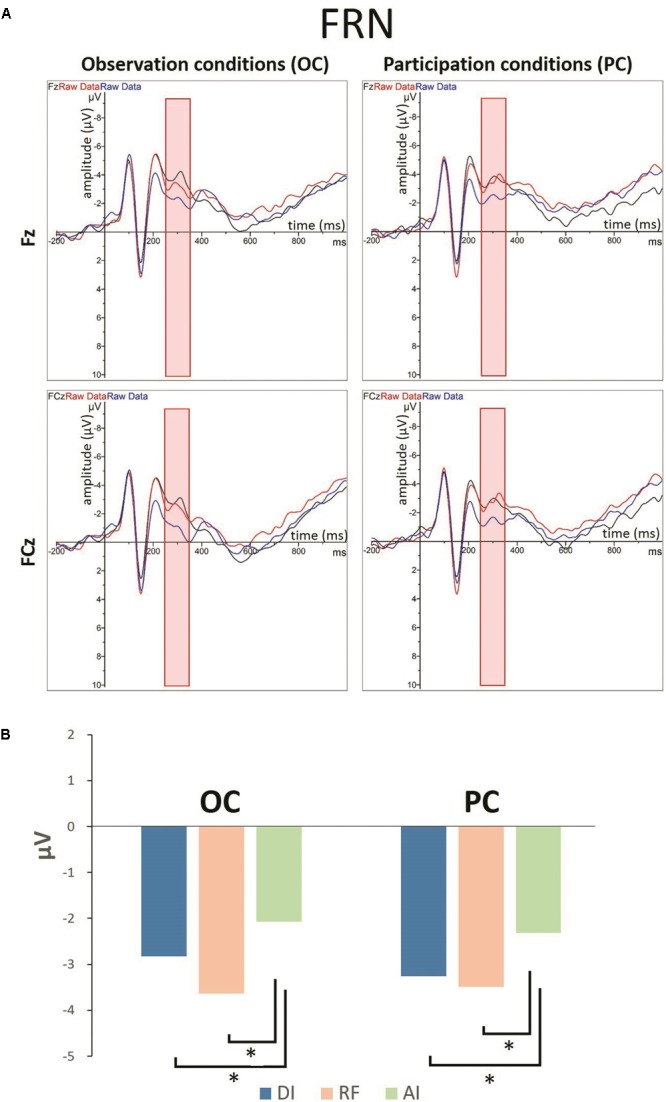
**(A)** Grand-averaged ERPs for LPP amplitudes in different situations from the Fz and FCz regions. **(B)** The bar graphs show the mean value of the FRN amplitude for each condition in Fz region. ^∗^*P* < 0.05.

Figure 5A, 6 show grand-averaged ERPs and topographic maps for FRN amplitudes in different situations. Similar statistical analyses were conducted for the grand-averaged ERP of FRN. The results revealed main effects for fairness [*F*_(2,50)_ = 9.935, *p* < 0.001, $\eta_{p}^{2}$ = 0.284] and electrode [*F*_(8,200)_ = 16.824, *p* < 0.001, $\eta_{p}^{2}$ = 0.882]. The main effect for condition was not significant [*F*_(1,25)_ = 0.913, *p* > 0.05, $\eta_{p}^{2}$ = 0.035]. *Post hoc* pairwise comparisons with Bonferroni correction showed the comparisons of AI (*M*_AI_ = -0.562, *SE*_AI_ = 0.826) with RF (*M*_RF_ = -1.804, *SE*_RF_ = 0.740) and with DI (*M*_DI_ = -1.555, *SE*_DI_ = 0.809) were both significant [*t*_AI,RF(25)_ = 3.819, *p*_AI,RF_ < 0.01; *t*_AI,DI(25)_ = 3.561, *p*_AI,DI_ < 0.01], whereas the comparison between RF and DI was not (see Figure 5B). The results indicate that the grand-averaged ERPs of FRN were larger for the DI and RF than for the AI distribution, regardless of condition. In addition, compared with the electrodes of Cz (*M* = -0.215, *SE* = 0.811), C3 (*M* = 0.613, *SE* = 0.638), C4 (*M* = 0.521, *SE* = 0.711), FCz (*M* = -2.012, *SE* = 0.902), FC3 (*M* = -1.071, *SE* = 0.759), and FC4 (*M* = -1.300, *SE* = 0.780), more pronounced FRNs were observed at Fz (*M* = -2.936, *SE* = 0.892), F3 (*M* = -2.489, *SE* = 0.819), and F4 (*M* = -2.873, *SE* = 0.819). Table 3 shows the mean amplitudes and standard error in each condition at FRN. The interaction effect between fairness and electrodes was also significant [*F*_(16,400)_ = 4.663, *p* < 0.01, $\eta_{p}^{2}$ = 0.882). DI and RF distribution to the coplayers elicited a more pronounced FRN than AI distribution at electrodes C3, C4, Cz, FCz, and FC3. Table 4 shows the significant *t*-test results for these electrodes in each condition at FRN. There was a similar tendency at other electrodes, but these were not statistically significant. The interaction effects of other variables were also not significant.

**FIGURE 6:**
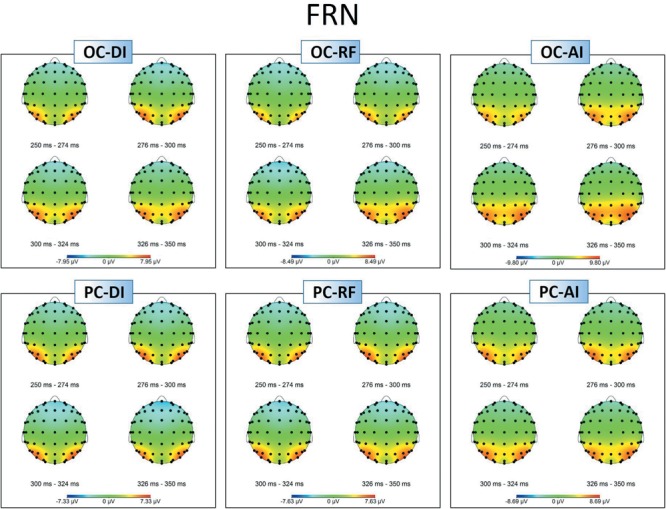
Topographic maps of the FRN amplitudes in different situations.

**Table 3:** Mean amplitudes (μV) and standard error in each condition at FRN (250–330 ms).

Electrodes	Observation condition			Participation condition		
	DI	RF	AI	DI	RF	AI
F3	–2.33 ± 0.92	–3.21 ± 0.80	–1.83 ± 0.92	–2.77 ± 0.86	–2.81 ± 0.75	–1.98 ± 0.91
F4	–2.75 ± 0.93	–3.39 ± 0.85	–2.16 ± 0.91	–3.18 ± 0.86	–3.23 ± 0.77	–2.52 ± 0.91
C3	0.64 ± 0.70	0.15 ± 0.67	1.56 ± 0.76	–0.06 ± 0.66	0.26 ± 0.61	1.13 ± 0.66
C4	0.41 ± 0.76	0.10 ± 0.77	1.70 ± 0.79	–0.12 ± 0.74	0.06 ± 0.72	0.98 ± 0.75
Fz	–2.83 ± 1.00	–3.64 ± 0.88	–2.08 ± 0.99	–3.26 ± 0.95	–3.49 ± 0.86	–2.32 ± 0.98
Cz	–0.35 ± 0.87	–0.65 ± 0.85	1.27 ± 0.94	–1.07 ± 0.85	–0.87 ± 0.81	0.38 ± 0.86
FCz	–2.05 ± 0.99	–2.62 ± 0.90	–0.77 ± 1.03	–2.64 ± 0.96	–2.65 ± 0.88	–1.36 ± 0.97
Pz	4.59 ± 2.72	4.49 ± 2.88	6.67 ± 2.82	3.53 ± 2.79	3.89 ± 3.19	5.34 ± 2.85
FC3	–1.00 ± 0.84	–1.61 ± 0.76	–0.21 ± 0.87	–1.63 ± 0.79	–1.39 ± 0.71	–0.59 ± 0.81
FC4	–1.28 ± 0.86	–1.76 ± 0.82	–0.34 ± 0.87	–1.72 ± 0.81	–1.73 ± 0.75	–0.97 ± 0.84

*DI, disadvantageous inequality; RF, relatively fair; AI, advantageous inequality*.

**Table 4:** Significant *t*-test results of electrode in each condition at FRN (250–330 ms).

	Observation		Participation	
Electrodes	condition		condition	
	*t*	*p*	*t*	*p*
C3				
DI_AI	–2.744	0.011	–4.789	0.000
RF_AI	–4.083	0.000	–2.723	0.012
C4				
DI_AI	–3.395	0.002	–3.897	0.001
RF_AI	–3.711	0.001	–2.843	0.009
Cz				
DI_AI	–3.870	0.001	–4.379	0.000
RF_AI	–4.193	0.000	–3.526	0.002
FCz				
DI_AI	–3.108	0.005	–3.702	0.001
RF_AI	–3.591	0.001	–2.940	0.007
FC3				
DI_AI	–2.334	0.028	–3.373	0.002
RF_AI	–3.672	0.001	–2.138	0.042

*DI, disadvantageous inequality; RF, relatively fair; AI, advantageous inequality*.

## Discussion

The main aim of this study was to investigate how the involvement of self-interest regulates empathic responses toward those who suffer unfair treatment. A related aim was to identify which component of empathy is more likely to be affected by self-interest. The electrophysiological data showed a decreased amplitude of the LPP when self-interest was involved. However, self-interest did not significantly affect the amplitude of N170. Disadvantageous inequality and relatively fair distribution to the coplayers elicited a more pronounced FRN than advantageous inequality distribution in both the OC and PC. Moreover, the behavioral data showed that the participants’ empathic responses were congruent with the coplayers’ emotional expressions in the OC, whereas in the PC their emotional responses were inconsistent with the coplayers’ expressions. These findings show that the participants’ empathic responses were aroused spontaneously when they observed the facial expressions of the coplayers at the early stage of emotional responses. However, self-interest modulated their empathy toward others at the late stage of responses.

At the temporal dynamics level, the neural encoding of facial expressions (reflected in the N170) was not affected by self-interest. Thus self-interest did not interfere with the emotional resonance with others. In other words, the results indicate that the early stage of empathy to economic inequality (i.e., emotional sharing) was not weakened by self-interest. However, the late stage of empathic responses (reflected in the LPP) showed differences between the OC and PC.

According to previous studies, there are three reasons for assuming that empathic responses toward others declines when self-interest is involved. Firstly, previous research has confirmed that LPP amplitude is associated with empathic responses, with statistical results showing a positive correlation. Participants displaying the greater LPP ‘pain effect’ said that they were more likely to experience empathic concern and to imaginatively transpose themselves into fictional social situations (Ikezawa et al., 2014). Our results indicate that the participants produced more empathic responses toward others in the OC.

Secondly, a series of studies found that LPP is associated with emotional regulation (Olofsson et al., 2008; Hajcak et al., 2010). A study found that when participants were asked to decrease the intensity of their emotional responses to unpleasant images, LPP amplitude was reduced (Moser et al., 2006). Foti and Hajcak’s (2008) finding further suggested that changing the meaning is indeed capable of modifying LPP responses to affective images (Foti and Hajcak, 2008). Based on previous studies, we consider that the amplitude of LPP can reflect the strength of empathy. If the participants have a stronger empathic response to the coplayers, they need greater emotional regulation which results in greater LPP amplitude. Similarly, weaker empathic responses could result in a smaller LPP amplitude. In our study, the amplitude of LPP increased in the OC and decreased in the PC. This suggests that the participants exhibited stronger empathic responses to the coplayers in the OC. In the PC, however, the participants’ concern about their own benefits might have weakened the empathic responses toward the coplayers, producing a lower LPP than in the OC.

Thirdly, previous studies have found that empathic brain responses are modulated by top–down control processes such as attention. Empathic responses can be reduced when attention is distracted away from others’ pain (Gu and Han, 2007). In the current study, the participants who were actively involved in the distribution of money were more concerned for their own interests; thus their attention shifted away from the coplayers’ outcomes while their empathic responses were reduced, reflected in the reduction in LPP amplitude.

Similarly to the LPP results, the behavioral results showed that participants in the OC rated themselves as feeling more unpleasant in the disadvantageous inequality situation than in the advantageous inequality and relatively fair situations. Meanwhile, they felt more unpleasant in the relatively fair situation than in the advantageous inequality situation. However, participants in the PC rated themselves as feeling more unpleasant in the advantageous inequality situation than in the disadvantageous inequality and relatively fair situations. Moreover, they felt more unpleasant in the relatively fair situation than in the disadvantageous inequality situation.

Additionally, participants in the disadvantageous inequality situation rated themselves as feeling more unpleasant in the OC than in the PC. In contrast, participants in the advantageous inequality situation felt more unpleasant in the PC than in the OC. There was no significant difference in the assessment of unpleasantness in the relatively fair situation between the OC and PC. These results suggest that fairness preferences and empathic responses depend on condition. The participants empathetically respond to others’ unfair payoffs only when their own interests are not involved.

These results are consistent with some previous studies, which revealed that individuals empathetically respond to others’ financial rewards. For instance, a handful of functional magnetic resonance imaging (fMRI) studies have demonstrated that the participants had greater reward region activation when they observed their friends or other people who were socially similar to themselves win at gambling (Mobbs et al., 2009; Molenberghs et al., 2014; Varnum et al., 2014). In contrast, when their self-interest was involved (i.e., when their low payoffs resulted from others’ high payoffs), the participants gave a higher unpleasantness rating to others’ high payoffs than to others’ low payoffs, suggesting that the advantageous inequality enjoyed by the coplayer was perceived as unfavorable by the participants, and vice versa. Thus the participants’ emotional responses were incongruent with the coplayers.

Evidence from ERP studies has shown that the temporal dynamics of empathy consists of an early affective arousal component followed by a late cognitive reappraisal and regulation component (Gu and Han, 2007; Decety et al., 2017). Our results suggest that self-interest reduces empathic responses toward others at the late stage of empathy. This implies that empathic brain responses are modulated by top–down processes. We speculate that the modification of empathic responses through self-involvement might be due to attention being drawn away from others’ emotional responses and toward the concern for self-interest. Research has confirmed that shifting attention away from emotional material results in reduced LPP amplitudes (Dunning and Hajcak, 2009; MacNamara and Hajcak, 2009; Thiruchselvam et al., 2011, 2012; Wangelin et al., 2011; Wiens et al., 2011; Nordstrom and Wiens, 2012; Paul et al., 2013; Wiens and Syrjanen, 2013). In addition, a series of studies showed that distraction yielded an attenuation of LPP magnitude in response to negative pictures and positive pictures (Thiruchselvam et al., 2011; Wangelin et al., 2011; Van Dillen and Derks, 2012; Paul et al., 2013; Schönfelder et al., 2014). Thus, as regards the current study, we speculate that participants’ attention could have shifted away from others’ outcomes to their own interests, resulting in reduced empathic responses that were reflected in the reduced LPP amplitude; i.e., distraction could have regulated their empathic responses. Although the studies mentioned above applied different experimental paradigms compared to the current study, the internal mental processing may be similar. Future research is needed to verify this speculation.

The ERP results also indicate that the disadvantageous inequality situation elicited a more pronounced FRN than advantageous inequality in both the OC and PC. Previous studies have shown that FRN is more prominent for stimuli with unfavorable as opposed to favorable outcomes (Gehring and Willoughby, 2002; Holroyd and Coles, 2002; Nieuwenhuis et al., 2005; Holroyd et al., 2006; Masaki et al., 2006; Hajcak et al., 2007; Yu et al., 2007; Walsh and Anderson, 2012). From this point of view, our study suggests that the participants perceived others’ frowns as unfavorable results at the early stage of empathy, regardless of whether they participated personally in the money distribution task. These results indicate that the participants’ initial emotional responses were congruent with the coplayers’, and therefore produced empathic responses. This is supported by the N170 results, which indicate that facial expression recognition was not affected by self-interest; thus emotions were aroused automatically. According to the two components of empathy theory, the empathic responses that take place at the early stage of empathy are elicited automatically by the perception of others’ emotional states. After the spontaneous empathic response toward others, their attention begins to drift away from others’ emotional states to their own interests, resulting in an attenuation of empathic response. These results further confirm that empathic brain responses are modulated by top–down processes such as attention. Concern about self-interest shifted attention away from others’ emotional responses and reduced empathic responses toward others at the late stage of empathy.

In summary, our results indicate that the participants exhibited empathic responses when others were treated unfairly. In the money distribution task, the participants’ empathic responses were aroused spontaneously when they saw the coplayer’s facial expression, regardless of whether their personal interest was involved, and especially at the beginning of their emotional responses. In the late stage of empathy, however, when self-interest was involved, the participants were more concerned about their own outcomes compared with others’ benefits, which reduced their empathic responses toward the coplayers. These results confirm that empathic responses are indeed regulated by the involvement of self-interest at the late stage of empathy. The participants showed a strong preference for self-gain. They preferred advantageous inequity for themselves, although the outcome might have been disadvantageous inequity for the coplayer. These findings are partially similar to the results of previous studies that have examined competitive situations; those results indicate that when individuals participate in a competitive task, they are most affected by their own outcomes. In comparison to strangers, participants’ empathic responses toward close friends were only salient when they did not directly participate in a gambling game (Leng and Zhou, 2010; Ma et al., 2011; Wang et al., 2014b). Research also suggests that the larger the extent of self-benefit involvement, the more likely it is that egoism will influence one’s affective response toward others’ monetary outcomes (Wang et al., 2014a). Our findings complement those studies; that is, in demonstrating on the contrary that people are not wholly concerned about personal interests. They can also be affected by other people’s emotional states, producing empathic responses at an initial stage.

Furthermore, empathic responses toward the coplayers were reduced when attention was diverted away from the coplayers’ outcomes. Therefore, we speculate that distraction cannot only regulate spontaneous emotions but also regulate vicarious emotions such as empathy. Empathy helps us to understand other people’s emotional states, motivates prosocial behavior, and facilitates social communication (Acevedo et al., 2012). However, professional workers such as counselors and doctors should not confuse their own experience with that of their clients (Luo et al., 2013); otherwise, it would lead to personal distress and detrimentally affect their well-being. In addition, high levels of empathic accuracy have been observed in individuals with psychopathic disorders, borderline personality disorders, and high levels of narcissism and anxiety, suggesting that a heightened awareness of, or sensitivity to, others’ internal states has the potential for negative outcomes (Simpson et al., 1999). An implication of our findings is that distraction is an effective means of regulating vicarious emotion, such as empathy. This has profound significance and important practical value for research in psychological counseling and clinical psychology.

## Limitations and Future Directions

Note that there are still some limitations to our current study. First, we only examined some healthy college students as participants in this research. The empathic responses of people with affective defects should be considered, and the differences between the temporal dynamics of empathy regulation by self-interest involved in healthy individuals and those with psychopathology should also be detected. We infer that the weakening of empathic responses toward others through concern about self-interest may be due to distraction. It is possible that regulatory processes in certain affective disorders may be characterized by distinct temporal dynamics. For instance, relative to low anxious individuals, those high in anxiety show a slowed disengagement of attention from threatening stimuli (Fox et al., 2001; Yiend and Mathews, 2001). Comparing healthy and psychopathological samples could illuminate the mechanisms behind the empathy regulatory impairments that underlie specific psychiatric disorders.

A second limitation is that the participants completed two types of money distribution task with the same gender-matched people. In daily life, we are in contact with people not only of the same sex but also of the opposite sex. Takahashi and his colleagues found that when the target person’s possession was superior and self-relevant (including the same sex), stronger envy and stronger anterior cingulate cortex (ACC) activation were induced (Takahashi et al., 2009). This implies that we may produce varying degrees of empathic responses to people of a different gender. Future studies should examine whether our findings are applicable to the opposite sex when they suffer from unfair money distribution.

## Author Contributions

JJ designed the experiments, analyzed the data, and wrote the manuscript. PL designed the experiments and wrote the manuscript. MZ, BL, YP, and JL collected the data. XZ designed the experiments and worked on the final version of the manuscript.

## Conflict of Interest Statement

The authors declare that the research was conducted in the absence of any commercial or financial relationships that could be construed as a potential conflict of interest.

## Supplementary Material

FIGURE S1 Grand-averaged event-related brain potentials (ERPs) for the N170 amplitudes in different situations from the P7 and P8 regions. OC, observation condition; PC, participation condition.

FIGURE S2 Topographic maps for the N170 amplitudes in different situations. OC, observation condition; DI, disadvantageous inequality; RF, relatively fair; AI, advantageous inequality; PC, participation condition.
