# Supplementary figures and images for: Self-Interest Induces Counter- Empathy at the Late Stage of Empathic Responses to Others’ Economic Payoffs
Source: Front Psychol. 2019 Feb 25;10:372. doi: 10.3389/fpsyg.2019.00372 (PMC6398428; doi:10.3389/fpsyg.2019.00372)

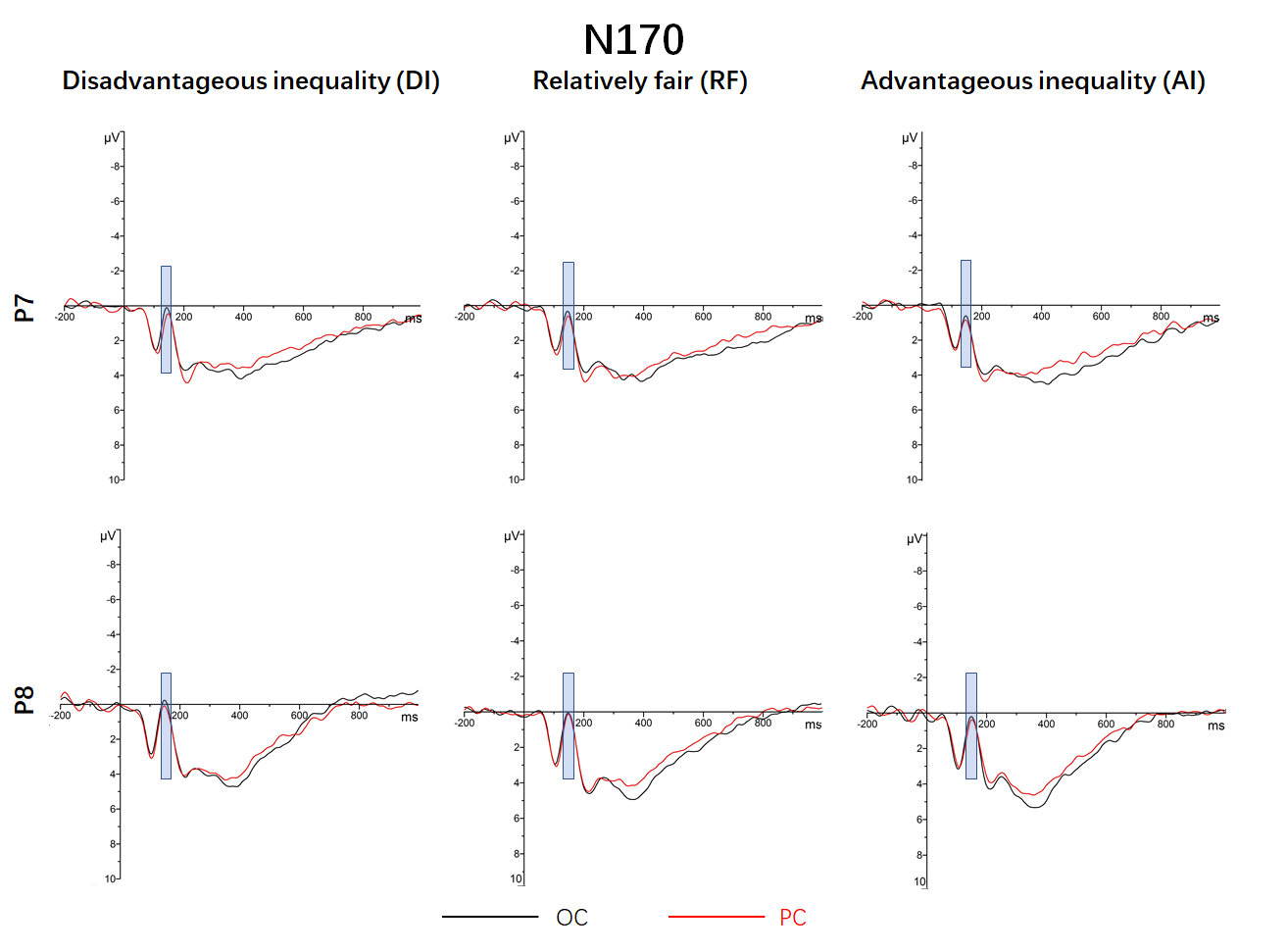

Supplement: FIGURE S1 — Grand-averaged event-related brain potentials (ERPs) for the N170 amplitudes in different situations from the P7 and P8 regions. OC, observation condition; PC, participation condition. [file Image_1.TIF]

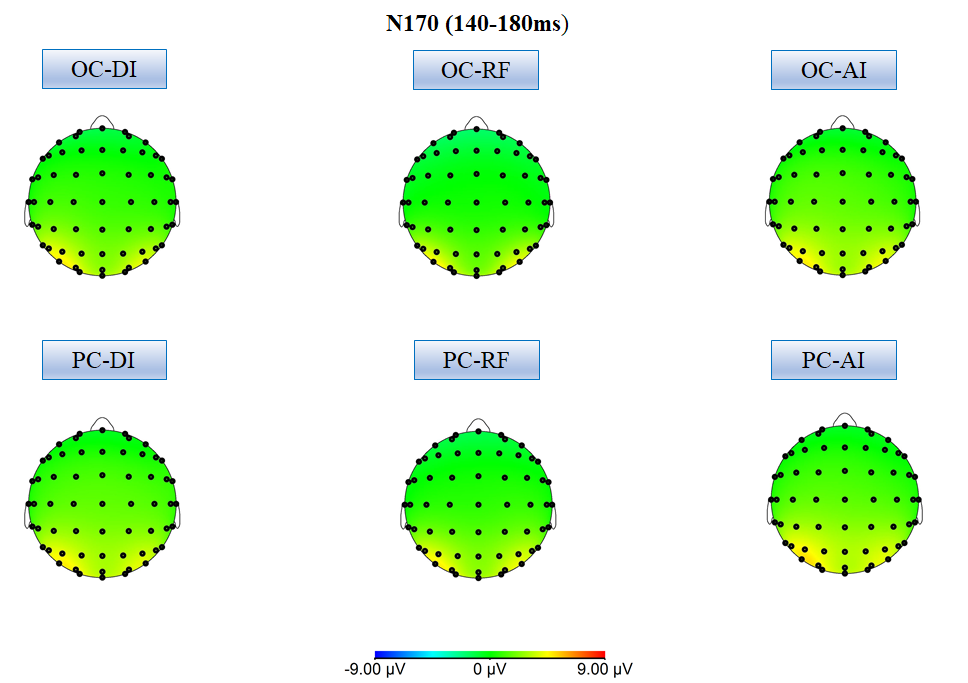

Supplement: FIGURE S2 — Topographic maps for the N170 amplitudes in different situations. OC, observation condition; DI, disadvantageous inequality; RF, relatively fair; AI, advantageous inequality; PC, participation condition. [file Image_2.TIF]
